# Medico-legal considerations in endodontics

**DOI:** 10.1038/s41415-025-8333-z

**Published:** 2025-04-11

**Authors:** Alyn Morgan, Callum Youngson, William McLean

**Affiliations:** 643706139754010723044 Director, U Dentistry Ltd, Ilkley, UK; Senior Clinical Teaching Fellow, School of Dentistry, University of Leeds, Clarendon Way, Leeds, LS2 9LU, United Kingdom; 457080896022727207443 Emeritus Professor, Liverpool Dental School, Faculty of Health and Life Sciences, University of Liverpool, L5 3PS, United Kingdom; 435563688494384037521 Professor of Endodontology, Glasgow Dental School, College of Medical, Veterinary and Life Sciences, University of Glasgow, Sauchiehall Street, Glasgow, G2 3JZ, United Kingdom

## Abstract

A major aim of endodontic care is to successfully treat acute and chronic pulpal and periapical disease and prevent recurrence. Consequently, the tooth can be rendered free of pain and subsequently restored to function and aesthetics. However, each of the stages of the treatment - from diagnosis through to review - can be complex and compromise the intended outcome. Occasionally, this can lead to medico-legal challenges, especially where a valid consent process has not taken place or has not been properly recorded. This paper reviews the key stages in providing care with respect to the clinician's skillset, the expected standard and discusses how the consent process can mitigate the risk of medico-legal interventions.

## Key points

- This paper considers the medico-legal risks involved in providing endodontic care.
- A number of strategies are proposed to help the clinician to mitigate these risks.
- Advice is offered to help clinicians who receive a complaint from a patient or their legal representative.

## Introduction

The United Kingdom (UK) and Ireland are now considered, anecdotally, to be the most dento-legally litigious countries in the world, with subtle differences between the constituent regions and countries, relating to cultural, social, legal and judicial differences. Society is also increasingly consumerist, with greater access to information via the internet, and some legal firms market a ‘no win-no fee' approach. Additionally, endodontics is a clinical discipline where litigation is common.^1^^,^^2^

To mitigate the risk of litigation, an appropriately documented, patient-centred consent process will help to defend the clinician's position. The growing availability of artificial intelligence (AI) transcribed clinical records and outputs may well significantly lighten the administrative burden associated with record-keeping and consent processes and allow an appropriate focus on treating the patient. However, it is worth bearing in mind that, while AI tools are also moving into radiographic assessment, diagnostic and prognostic areas (which can help to inform a clinician's judgement), the operator themselves will be held responsible for the decisions that they make when providing treatment.

Dentists' concerns regarding regulation and litigation are well-recognised^3^^,^^4^^,^^5^ and ‘defensive' approaches can be adopted, including avoiding specific forms of treatment due to a dentist's concerns about their competence and confidence^6^ (see later). This can lead to increasing referrals to more competent or confident practitioners (e.g. specialist endodontists) but also to the referring dentist deskilling due to ‘professional disuse atrophy', as well as congestion of the referral system and overburdening of the limited number of specialists. An effective healthcare system therefore requires strategic governmental planning to ensure that there are sufficient numbers of generalists and specialists in training and practice to meet the needs of the population. It is also important, medico-legally, not to represent one's clinical credentials in a misleading way, with the term ‘specialist in endodontics' having a specific, regulatory, definition.

Some inappropriate referrals may take place due to a misunderstanding of the standard of care that should be provided, and knowledge of the expected standard may improve the confidence of the dentist to provide treatment within their competence and identify those cases which genuinely require referral.

## Standard of care

Initial guidance on the expected standard of care was adopted in the late 1950s with the ‘Bolam test',^7^ where, essentially, treatment provided should correspond with that expected from a reasonable clinician. This was strengthened in the late 1990s by the ‘Bolitho test',^8^ where treatment decisions had to be supported by a reasonable body of opinion. The expected standard of care will therefore depend upon who provided the treatment, as well as when it was carried out and will be slightly different for those practising as a specialist, rather than a general dental practitioner (GDP). To help the profession codify the expectations for the GDP, the Faculty of General Dental Practitioners published the *Standards in dentistry* in 2006 (updated in 2016).^9^ This document recognised that ‘expected standards' documents could be misinterpreted by regulators or legal entities as ‘required standards', and so categorised them for each clinical area (including endodontics) as ‘aspirational', ‘basic', or ‘conditional'.^9^ For endodontic specialists in the UK, expectations would be to comply with the standards embedded in *Good endodontic practice*.^10^

### Inherited cases

A comprehensive dental history is required, including whether the patient received orthodontic treatment or suffered dental trauma.^11^ A complication when assessing the standard of care of previously provided endodontic treatment is when it was provided originally. In the past, some treatments taught in dental schools (e.g. single silver cones) were considered ‘reasonable' but are now unacceptable. When a patient received endodontic care that was acceptable at that time (in the absence of clinical or radiographic evidence of periradicular pathosis, and alongside documented discussion with the patient), it would be reasonable for the current dentist to monitor the tooth using contemporary methods. Any retreatment would, however, be expected to conform to the current, not previously accepted, standard.

What approach should be adopted with a radiographically ‘short' root filling in the absence of overt (conventional) radiographic periradicular changes or clinical symptoms? While some litigants may claim that anything short of the probable radiographic apical constriction is deficient, the European Society of Endodontology's quality guidelines^12^ do not state an arbitrary distance from the radiographic apex, but instead observe that ‘no space between canal filling and canal wall should be seen. There should be no canal space visible beyond the endpoint of the root canal filling'. This allows for the variety of periapical morphologies and supports a biological, rather than technical, approach to considering whether retreatment is indicated. Even putting aside anatomical variants that may lead to an apparent technical shortfall in existing treatment, biologically based decision-making is important. Providing retreatment based upon technical deficiencies alone is not sufficient and wider consideration must be made to the risks associated with retreatment in the absence of signs and symptoms of disease.

### Newly arising cases

A key consideration when providing care is to gain valid consent, where the patient is appraised of all the facts that they would personally consider to be important before committing to treatment - a process that has become known as the ‘Montgomery test'.^13^ After making a diagnosis, it is important to discuss with the patient the range of treatments which may be offered to treat the presenting condition. Although this paper is considering the medico-legal aspects of endodontic treatment, the clinician should discuss with their patient, given the specific circumstances, a range of reasonable alternative treatments which could include, in some cases, extraction with leaving the space, or restoring the space with some form of a prosthesis, with the likely benefits and drawbacks, longevity and cost of each option.

#### Vital pulp therapies

There is a disease continuum, from mild pulpal inflammation to frank necrosis and infection, with the classic signs and symptoms associated with periradicular pathosis. Where possible, treatments should be focused on regaining pulpal health by appropriate caries/dental trauma/pulpal management. Longitudinal, contemporaneous, clinical records (radiographs and notes) are very useful to demonstrate the process used in clinical decision-making. Recording any changes in diagnosis and summaries of discussions with the patient, regarding their wishes, demonstrates an ongoing consent process that helps mitigate complaints and greatly increases defensibility should litigation arise.

Improvements in our understanding of pulp biology, the pulpal response to insult and development of bioactive materials have led to the potential to maintain pulps in even fully mature, multi-rooted teeth that were previously considered to have ‘irreversible pulpitis'^14^^,^^15^ (more accurately, severe pulpitis).^16^ While there are currently few high-quality, long-term clinical studies to demonstrate the likely success rate of these forms of treatment^17^ (which complicates demonstrating valid consent has been gained), there is a growing body of evidence that supports vital pulp therapies as an alternative to conventional root canal treatment^18^^,^^19^ and - if communicated to the patient - does not preclude provision of the treatment in appropriate cases.

#### Non-surgical root canal treatment

Communication of the clinical diagnosis is crucial to gain valid consent, as the patient needs to know why, as much as how, a treatment will be provided. This also allows the clinician to recognise factors which may complicate cases e.g. by identifying where an acute exacerbation arising from treatment is more likely, with a warning to the patient accordingly.

The discussion (with appropriate clinical records) of the risks and probable benefits, specifically associated with non-surgical root canal treatment (NSRCT) is also an important part of the consent process. One important aspect is the likely longevity of the proposed treatment. While this is less predictable in vital pulp therapies, a number of studies demonstrate that a very high percentage of NSRCT-treated teeth are retained long-term after treatment; although, some of the prognostic factors depend on the familiarity of the operator with the techniques they are using and the complexity of the case.^20^^,^^21^

## Complexity assessment and consent in endodontics

An essential part of the endodontic treatment planning process is a definitive diagnosis and a thorough analysis of all the factors that contribute to the complexity of the intended treatment. Once completed, this complexity assessment will be at the core of the consent discussion with the patient as it informs *inter alia* who is best placed to undertake the treatment, the likelihood of technical and biological success, the potential longevity of the tooth post-operatively, and how this might compare with alternative treatment suggestions. It is then possible to complete the plan of treatment and have a meaningful discussion with the patient about their options to gain valid consent.

Complexity and risk assessment should take into consideration factors that will impact on the treatment process and its outcome.

Complexity assessment is the consideration of all the potential technical barriers to completion of an endodontic procedure, for example, root canal curvature, extent of canal sclerosis/calcification and physical access to the tooth.

Risk assessment is broader, considering the risks and benefits to the patient, combining complexity assessment with other factors which may impact on overall treatment outcome, such as the size of any periapical lesion, or the structural integrity and periodontal status of the tooth. Risk assessment would also include the patient's general health and how this might impact the execution of the treatment and its potential outcome. Records should be made of other factors, such as any medication the patient takes (e.g. bisphosphonates, monoclonal antibody medication or anti-coagulants), which could favour root canal treatment over extraction in a treatment plan.^22^^,^^23^

A number of complexity assessment tools are available (Box 1)^24^^,^^25^^,^^26^^,^^27^^,^^28^ which offer a structured approach to complexity and risk assessment, ensuring a systematic approach to choose the most appropriate treatment for the given circumstances. Increasingly, assessment tools are available digitally, making them more efficient and allowing the outcome to be included easily in the patient's clinical records. Although these systems provide guidance, there is a requirement for subjective operator input, particularly around radiographic interpretation, and the operator needs to have a clear understanding of the features they are recording from a radiograph. With rapid growth in the use of AI for medical and dental diagnostics, it is likely that automated assessment tools will become available which use machine- and deep-learning models to assess the complexity of treatment and offer a prediction on treatment outcome from pre-operative radiographs.^29^^,^^30^^,^^31^ Currently, however, complexity assessment predominantly relies upon the operator assessing a pre-operative periapical radiograph (ideally using one of the assessment tools for guidance) and correlating the outcome of this assessment with their own skillset, before communicating these aspects to their patient and agreeing a plan of treatment. Where the clinician has concerns about the quality of information available from a plain film, this should be discussed with the patient and a decision made as to whether further investigations are required before making a final treatment decision.

Limited field of view (FOV) cone beam computerised tomography (CBCT) is increasingly used in endodontic diagnosis and assessment. The ability to assess root canal curvature in all three anatomical planes, have a clearer understanding of the number of canals present and, at higher resolutions, identify sclerosed canals that may not be visible on periapical radiographs, are some of the ways in which CBCT offers superior diagnostic information. The current European Society of Endodontology position statement on the use of CBCT in endodontics^32^ states it ‘should only be considered after a detailed clinical examination, including conventional radiographs, has been performed…potential benefits as well as potential risks must be discussed…CBCT must be used judiciously' and ‘a small FOV CBCT examination should be considered if the additional information…is likely to aid diagnosis and treatment planning and/or enhance clinical management'.

To use CBCT, there is a requirement for the user to have undertaken the necessary training to order and, if appropriate, report on scans. While this is the minimum requirement for dentists using CBCT, there is also skill required in interpreting scans to accurately assess for endodontic complexity. Dentists who intend to regularly use CBCT for endodontic treatment are advised to ensure they have received appropriate training in this aspect of reporting.^33^

Box 1 Complexity tools used in endodontic assessment

- Clinical standards for restorative dentistry, NHS England^24^
- EndoApp^25^
- AAE Case Difficulty Assessment Form^26^
- Dutch Endodontic Treatment Index and Endodontic Treatment Classification^27^
- E-CAT^28^

### Radiographic complexity assessment

Several complexity assessment tools split complexity into three categories. These refer to the training and expertise which is considered necessary to successfully treat cases, with simple cases capable of being undertaken by a GDP, the second category by dentists with enhanced postgraduate training in endodontics, and the third by endodontic specialists.^24^ A UK study noted 40% of cases fall into the first, 32% the second, and 28% into the third category.^34^ Failure to follow the guidance of the assessment tools and undertake treatment outside the clinician's particular skillset leads to increased procedural complications^35^ and could lead to sub-optimal treatment outcomes which, if litigation was pursued, could be difficult to defend successfully.

## Case complexity - radiographic assessment

Key elements of case complexity include root canal curvature, canal appearance (calcification/sclerosis/resorption) and apical condition (resorption, iatrogenic damage).

### Root canal curvature

Even with contemporary instruments, root canal curvature is a major factor in case complexity. The angle, but more importantly the radius of curvature, are important to consider. Most guidelines suggest that an angle of curvature greater than 45 degrees warrants specialist treatment. However, rather than one specific angle being a cut-off point, the non-specialist should consider their own competence and confidence in tackling less straightforward cases. They should record their findings accurately and then decide (for example, in a case where the canal has a large radius of curvature and the clinician is experienced in using modern nickel-titanium endodontic files), whether they are likely to be able to undertake successful canal preparation without procedural errors. They should then discuss their decision with their patient to gain valid consent, rather than automatically refer the case to a specialist.

**Fig. 1:**
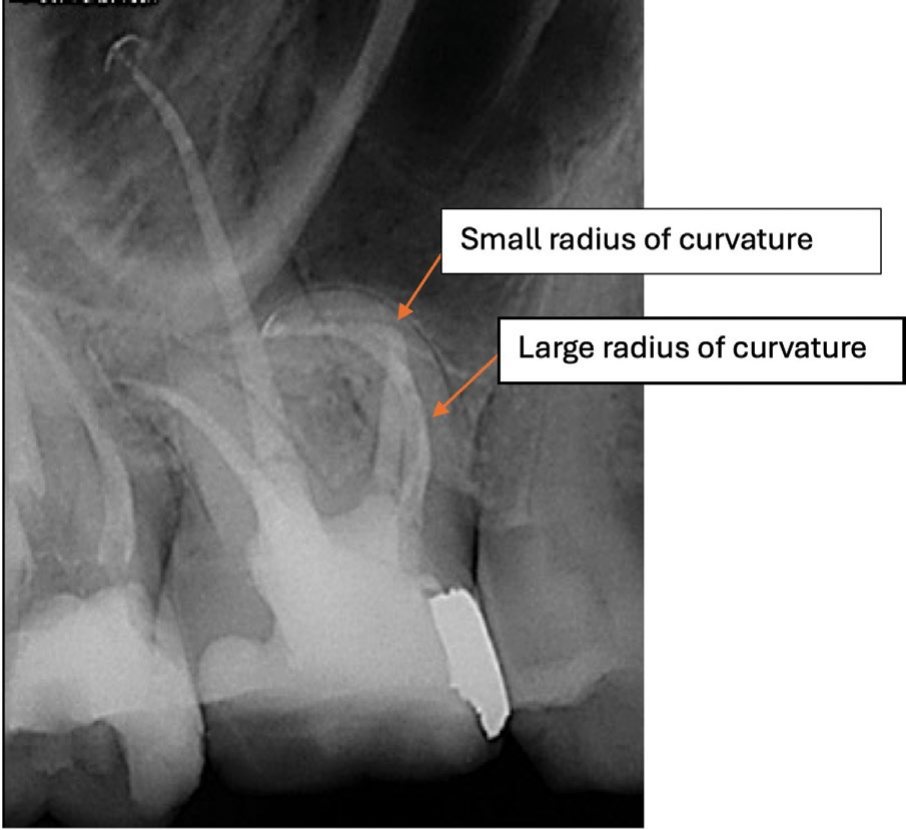
Plain film radiograph illustrating a large and small radius of curvature

An entirely different situation arises where a canal demonstrates a large angle of curvature with a small radius of curvature (Fig. 1). This is a significantly more challenging situation with increased risk of canal ledging, blockage, or file fracture and would suggest that specialist-level endodontic care is required.

**Fig. 2:**
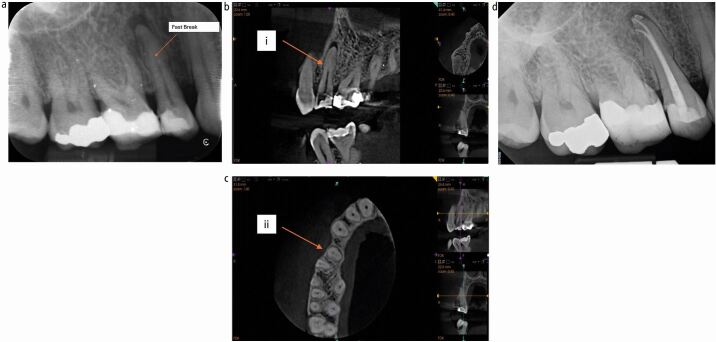
a) Probable canal bifurcation in the plane of the radiograph demonstrating a ‘fast break'. b, c) CBCT of tooth: fast break (i), canal bifurcation evident at mid-root level (ii). d) Completed RCT showing (obturated) bifurcated canal

**Fig. 3:**
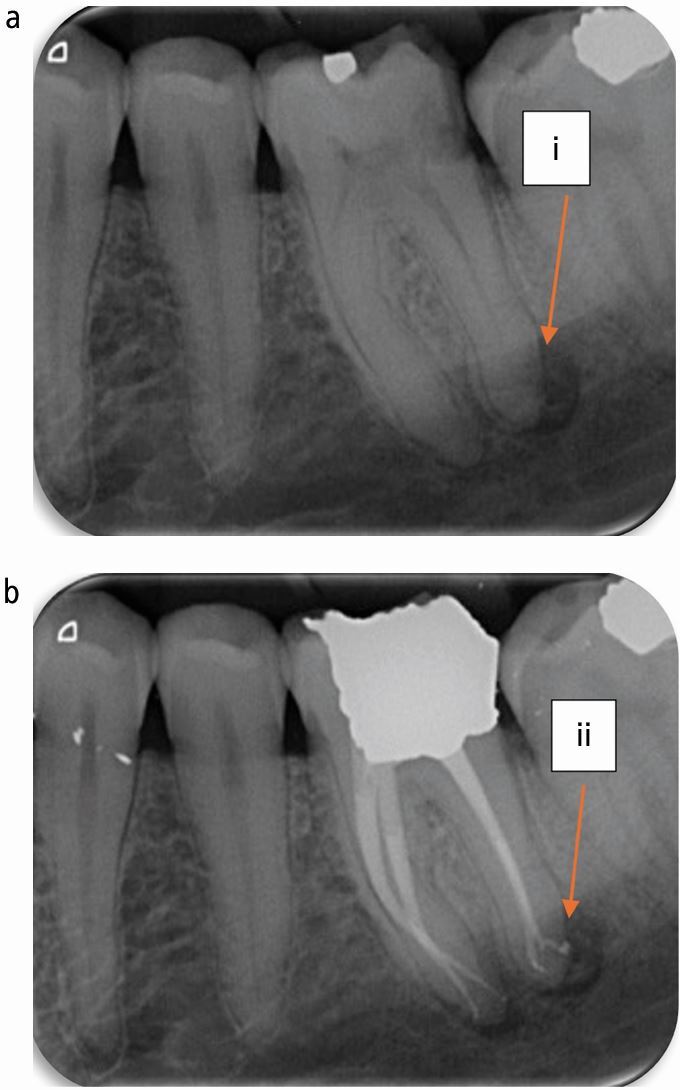
Position of the periapical lesion indicating the position of the portal of exit of the canal. a) Periapical radiolucency lateral to the radiographic apex of the root (i). b) Portal of exit of canal lateral to the radiographic apex and central relative to the periapical radiolucency (ii)

Assessing root canal curvature from radiographs can be difficult due to the resolution required to see the entire length of the canal and the multi-planar nature of the curves which, when viewed on a periapical radiograph, can cause the canal to apparently disappear. This ‘fast break' appearance is usually a sign of canal bifurcation^36^ or an acute curvature, often into the plane of the radiograph (Fig. 2). However, CBCT can offer a more predictable assessment method when a standard periapical does not offer clarity over the canal path (Fig. 2). Even without CBCT, there are visual clues on a periapical radiograph that can give the operator an indication of acute curvatures present; by studying the position of the periapical lesion, it is feasible to assess the position of the portal of exit of the canal and effectively trace its position in the root (Fig. 3).

In cases where the complexity assessment (based on a full clinical and conventional radiographic examination) suggests that specialist-level treatment is required, an appropriate referral should be discussed with the patient, or an alternative form of treatment considered.

## Surgical endodontics

Clinicians should reflect carefully upon their reasons for recommending periradicular surgery^37^ e.g. perforation repair or root-end-resection and retrograde filling, as well as their competence and confidence in performing these techniques. As well as considering the clinician's experience, many medico-legal cases in this field deal with whether guidelines were followed and whether there was an appropriate assessment of an original root canal treatment which was first provided or retreated to restore apical health, before surgical intervention. Once again, the patient must be fully informed to give valid consent to the procedure being provided, and this includes the offer to refer the patient to a specialist endodontist for this care.

## Electronic apex locators

Early models were rather unpredictable but the most recent generations of electronic apex locators (EALs), when used in accordance with the manufacturer's instructions, reliably and (medico-legally) defensibly determine the working length of the root canal.^38^^,^^39^ Their use can also: a) reduce the radiation dosage by eliminating the need for a diagnostic length radiograph; and b) indicate if a lateral perforation has occurred. However, if it is not possible to determine working length reliably using EALs, then a working-length radiograph should be exposed and, in the absence of a working-length radiograph, a mastercone/cone-fit radiograph may be advised, to demonstrate that the canal has been appropriately instrumented to the full length of the canal space. If any concerns are then noted, this allows the opportunity to discuss these and any remedies or alternative treatments with the patient before obturation.

## Perioperative pain and complications

### Endodontic emergencies

Pain of endodontic origin can arise before, during, or following completion of treatment. Prior warning of the risks of mid- or post-treatment pain (as a normal part of the consent process) and how this could be managed will reassure the patient that the clinician is knowledgeable and prepared for this eventuality, subsequently reducing the risk of the patient raising a complaint or seeking litigation successfully. Abbott^40^ recommends that these commonly arising, endodontic emergencies are managed using the principles of the ‘3-D's - ‘diagnosis', ‘definitive dental treatment' and ‘drugs' - in that sequence. Diagnosis forms the crucial first stage and, when this has been determined, it is important to discuss this with the patient so that they understand why they are in pain and the best way(s) to treat it. Many patients seek antibiotics as a first-line treatment, but only infection will respond (eventually) to antimicrobial therapy and anti-inflammatory drugs can only reduce pain associated with inflammation.

### Complications

While not required in all territories, the principles of ‘the duty of candour' that exists in the UK in both statutory^41^ and professional^42^ forms sets a good ethical start-point for managing complications after a procedural error has taken place. The General Dental Council (GDC) guidance^42^ notes:

- ‘This means that healthcare professionals must: tell the patient…when something has gone wrong; apologise to the patient…'.

Contrary to popular opinion, apologising is not admitting liability^43^ but can often help to defuse a difficult situation by the clinician displaying empathy and honesty, reducing the likelihood of a subsequent complaint.

In some circumstances, it will be immediately obvious to the patient that an untoward incident has taken place e.g. if a hypochlorite accident arises.^10^^,^^44^ Where a patient has been informed of the benefits of the use of sodium hypochlorite, but also the risks if it extrudes outside the root canal system (with a protocol to manage this eventuality), the clinician will be able to observe that they followed a reasonable body of opinion in using this irrigant^10^ but that an unforeseen complication occurred. An open discussion with the patient as to how the situation arose, with an appropriate apology, may not avoid litigation to compensate the patient appropriately (as significant soft-tissue damage can arise)^45^ but would reassure the regulator that the accident was not of itself evidence of unprofessional behaviour.

More often, a complication will not be obvious to the patient (e.g. a fracture of an instrument within the canal, lateral perforation of the root) or even immediately to the clinician (e.g. extrusion of the root-filling material into the periapical tissues). While these can be frustrating or embarrassing for the dentist, the ‘duty of candour' is important to fulfil their ethical duty to the patient, who is kept informed of the progress of their treatment and involved in the ongoing consent process. In the case of dealing with a fractured instrument and the decision to either leave it *in situ,* attempt removal, bypass it, or refer to a specialist, the clinician can demonstrate they have complied with the GDC duty of candour guidelines (and acted in the best interests of the patient) to:

- ‘Offer an appropriate remedy or support to put matters right (if possible); and explain fully…the short- and long-term effects of what has happened'.^42^

## Restoration of the root-filled tooth

Although slightly beyond the scope of this article, each tooth to be endodontically treated should be assessed for the quality and quantity of the remaining coronal structure before root-filling to determine whether it is restorable, and whether a simple or more complex restoration will be required afterwards to restore aesthetics, function and maintenance of a microbial seal.^46^ It is important that all decisions made are discussed with the patient and a record made of these.

## Defining competence in endodontics: beyond procedures

Competency is a concept embedded within dental education which describes the knowledge, skills and professional values of someone ready for beginning independent practice.^47^ To be deemed competent, an individual should understand the rationale behind clinical decisions, uphold professional values and have the capability to address the dental needs of a broad range of patients.^48^

Principle seven of the *Standards for the dental team*^6^ states one ‘should only deliver treatment and care if you are confident that you have had the necessary training and are competent to do so'. Failure to do so therefore presents a medico-legal risk but no guidance is given to specific competencies required in clinical fields. However, the American Association of Endodontists (AAE) has developed a competency framework^49^^,^^50^ detailing competencies in specific domains, including diagnosis, treatment planning, treatment provision, and prediction of outcome and post-treatment evaluation. The knowledge and skills required by all practitioners for competence in the domains are outlined, intended to be used in conjunction with case-difficulty assessment guides (see earlier) to ensure appropriate referral is made if the difficulty exceeds the practitioner's ‘experience and comfort'.

Competence in dentistry goes beyond technical prowess in performing specific procedures and the AAE documents do not merely list technical skills but also, among other aspects, incorporate critical application of knowledge, experience, ethical judgement and patient communication. These ultimately impact upon the capacity of the clinician to provide patient-centred care that minimises risk and maximises outcomes. It is important to recognise how one's own training aligns with these competencies and work within them.

Maintaining competency is essential as there is continuous evolution within the field of endodontics due to biological and technological advancements and evidence-based updates in practice guidelines. Thus, there is a need for ongoing engagement in the form of continuing professional development and, potentially, with formal postgraduate qualifications.

## Building and sustaining confidence: a nuanced perspective

Confidence in endodontic practice impacts upon patient safety and practitioner decision-making profoundly. Confidence has a ‘golden mean', with the extremes being arrogance and self-doubt, where overconfidence can lead to increased risk-taking. The importance of self-awareness cannot be overstated, as inappropriate handling of complex cases, such as those requiring surgical endodontics or management of resorption, could be the basis for claims, where lack of self-limitation could be cited as a contributing factor to patient harm. Under-confidence can cause unnecessary referrals, which may delay patient care. In endodontics, confidence is often linked to procedural familiarity, particularly in high-risk or high-stakes situations, such as retreatments and management of cases of higher complexity (as outlined previously). It has been established that there is a positive relationship between postgraduate education and a perceived increase in confidence of clinicians^51^ and this manifests itself in better communication and clinical skills.

It is now common for clinicians to seek to engage with postgraduate educational offerings provided by universities and the Royal Colleges, among others.^52^ There have also been advances in career pathways available to general dentists, with the advent of the ‘dentist with extended skills'.^53^ Not only does this training enhance care provision in a practice setting when aligned with an individual's competence and complexities of the case, but it also has a profound effect on the confidence of practitioners to deliver endodontic care, which may subsequently have a positive impact upon medico-legal risk.

## Mentoring

Involvement in the formal mentoring of colleagues is an important and rewarding professional undertaking. ‘Mentoring' is a term often used loosely within the dental profession; although, there is a specific definition and guide to ethical practice.^54^

Where a mentoring relationship involves discussion of clinical cases and complications arise, a mentee may suggest that they were working under the mentor's guidance, and liability for any harm that has arisen lies partially with the mentor. It is therefore important that, in any such relationship, it should be made clear at the outset that the mentor's purpose is to support the mentee's general development but that clinical advice offered is not guidance to the treatment of any specific patient. In an unpaid mentoring situation, any claim of joint liability is unlikely to be successful, as the mentee will be an appropriately qualified practitioner with their own registration. They will also have gained the patient's consent to the proposed treatment (and so own ‘the duty of care').

However, if the mentor is also acting as a clinical supervisor (particularly in a paid role), the situation could become more complicated, especially if it can be shown that the mentor has given clinical advice related specifically to the case in question (or that this advice formed part of the consent process). Furthermore, ethically, if the mentee's competence is being assessed by a paid mentor, care should be taken to avoid the perception of a conflict of interest, if additional mentoring is suggested to improve the mentee's performance. Where a clinical supervisory role forms part of the mentoring process, it is recommended that the mentor's indemnity organisation or insurer is informed.

## Informal advice

‘Remote consultations' increased as a consequent of COVID-19 restrictions. The technology to send encrypted messages and images has developed to the point where it is now easy to send (anonymised) images of radiographs to colleagues to seek their opinion. While peer-discussion on anonymised cases has always been commonplace, specialists particularly need to be aware that, if a generalist sends them a radiograph or brief outline of a case electronically for a ‘quick word of advice', they could be considered to have offered a specialist opinion should any legal action take place thereafter. However, if there was clearly no ‘referral' arrangement and no payment was received for the advice, which was of a general or technical nature based on limited information rather than specific clinical advice on how to treat a given patient, this would not constitute a specialist opinion.

## Dealing with cases/complaints

### Processes

The GDC make it a requirement of registration that the clinician holds appropriate indemnity or insurance and has issued guidance on what questions clinicians should ask before choosing their cover.^55^ Within some jurisdictions, it is also a requirement that each dental practice has a complaints procedure that is readily available for patients to use, and that the complainant must receive a prompt and constructive response.^6^ If the complaint concerns money, the clinician needs to consider how much time, effort and emotional energy will be spent trying to recover or retain payment, rather than resolving the dispute without necessarily accepting liability. It is, however, paramount that the clinician informs their indemnity organisation at a very early stage to gain advice and medico-legal support (and access to wellbeing support, where the cover provides this).

### Personal consequences

In the UK, dentists can expect, statistically, to be threatened with litigation twice in their practising lifetime. Although the likelihood is small, the threat is often highly significant, as professional and personal identities are closely intertwined. One study has noted the highly stressful nature of a GDC investigation, leading to dentists leaving the profession, and over one-quarter of respondents considering suicide.^56^ It is important therefore that when a complaint is received, that the clinician seeks support by:

1. Talking to their work colleagues, friends, family (and where appropriate their faith leaders) to avoid becoming isolated, and
2. Seeking professional help in the form of their indemnity/insurance organisation and receives appropriate counselling or medical intervention to maintain their wellbeing during the investigation process and beyond.

## Conclusion

It cannot be emphasised enough that concise but precise clinical records of the assessment, diagnosis, treatment planning and consent process, alongside provision of treatment to the current standard, with discussion of complications as they arise, in addition to ensuring that adverse outcomes are defensible,^57^ provides a significantly ethical, transparent and positive working environment for the whole team.

Contemporary techniques and an ever-evolving endodontic armamentarium mean that success rates for treatment and the possibilities to offer predictable solutions are greater than ever before. While this article discusses the medico-legal challenges associated with providing endodontic treatment, it should be recognised that with the proper risk assessment, training and mentoring, practitioners can become comfortable and confident in providing treatment to a high standard for the benefit of their patients.
